# Could the connectedness of primary health care workers involved in social networks affect their job burnout? A cross-sectional study in six counties, Central China

**DOI:** 10.1186/s12913-020-05426-9

**Published:** 2020-06-18

**Authors:** Yiqing Mao, Hang Fu, Zhanchun Feng, Da Feng, Xiaoyu Chen, Jian Yang, Yuanqing Li

**Affiliations:** 1 School of Medicine and Health Management, Tongji Medical College, Huazhong University of Science and Technology, Wuhan, 430030 Hubei China; 2 Research center for Rural Health Services, Hubei Province Key Research Institute of Humanities and Social Sciences, Wuhan, 430030 Hubei China; 3 The First Affiliated Hospital of Zhengzhou University, Zhengzhou, 450052 China; 4 School of Pharmacy, Tongji Medical College, Huazhong University of Science and Technology, Wuhan, 430030 Hubei China

**Keywords:** Primary health care workers, Connectedness, Social networks, Job burnout

## Abstract

**Background:**

This study aimed to reveal the effects of the connectedness of primary health care (PHC) workers in social networks on their job burnout.

**Methods:**

Cross-sectional survey data of rural PHC workers in China were analyzed. A total of 663 respondents were enrolled. Chi-square and cumulative logistic regression were used to determine the effects of the connectedness of PHC workers in social networks on their job burnout.

**Results:**

PHC workers in rural China had high levels of emotional exhaustion (24.1%), depersonalization (15.7%), and lack of personal accomplishment (34.7%). More than half of the participants were in the middle connectedness level in terms of their advisory (70.4%) and friendship (70.3%) networks. The degree of emotional exhaustion seemed to increase when participants had a low connectedness in their friendship networks (*β* = 0.769, 95% *CI* = 0.080–1.458, *P* = 0.029). Respondents with the middle level of connectedness in advisory networks had higher levels of depersonalization (*β* = 0.739, 95% *CI* = 0.130–1.348, *P* = 0.017) and lack of personal accomplishment (*β* = 0.583, 95% *CI* = 0.111–1.055, *P* = 0.015) than those with the high degree of connectedness in advisory networks.

**Conclusions:**

The connectedness of PHC workers in social networks influenced their job burnout. Thus, organizations should establish an informal communication platform and information feedback mechanism, promote and manage friendship networks, and help PHC workers overcome emotional exhaustion. Managers should also encourage individuals with a high level of connectedness in advisory networks play the role of “opinion leader” so that they can help others mitigate burnout.

## Background

Given the theme of the 72nd World Health Assembly (“From primary health care (PHC) to universal health coverage and sustainable development goal”), PHC has played an important role in implementing universal health coverage and achieving a healthy strategy in China. Healthcare workers are the first resource for PHC services [1]. However, the shortage of PHC workers is a global problem, and such concern is especially serious in rural areas [2]. In China, only 1.8 physicians per 1000 people were available in rural areas in 2018, a figure equivalent to only 45% of their counterparts for urban areas [3]. The shortage of personnel greatly affects the quality of PHC services. In addition to income level and career development prospects [4], job burnout is an important factor that affects the loss and shortage of rural PHC workers. Rural PHC workers commonly face job burnout, a situation that may contribute to the high staff turnover [5]. Accordingly, job burnout can increase the shortage of rural PHC workers. Burnout can also affect an individual’s physical and mental health and cause lower personal performance [6]. All of these can lead to the high instability of a PHC network.

Job burnout is a kind of sub-health state of chronic stress that is associated with limited resources, abilities, energies, and interests in long-term work. Furthermore, it involves emotional exhaustion, depersonalization, and lack of personal accomplishment [7]. Burnout is a global occupational hazard among PHC workers [5]. PHC workers experience burnout due to relatively less work resource, lower wages, and worse career development opportunities compared with health care workers in general organizations [8]. Previous studies also revealed that individual factors (such as age, gender, educational background, work experiences [years], monthly income, and daily work hours) can influence the job burnout of PHC workers [9–11]. Moreover, job burnout of different professional PHC workers vary. Xu reported that doctors had higher level of burnout than nurses, pharmacists, and other technicians of the PHC network [8].

In China, the unique health care service model in rural areas may lead to other determinants of job burnout. The service model of rural PHC workers had changed greatly, from providing services independently to working in teams since the implementation of new health care reform in 2009. Presently, most rural PHC workers who provide services take the team work for residents, such as establishing family physician groups [12]. The team work model generates more connections with others in the organization in comparison with independent working methods. Therefore, an interpersonal relationship in the team work mode can be more critical when working in a cooperative way, and such a relationship plays an important role in causing the job burnout of PHC workers. At present, some research have revealed the relationship between interpersonal relationship and job burnout. As Hayes B’s a review, the relationship of nurses with physicians had the highest negative influence on job burnout [13]. Sun JW confirmed that interpersonal relationships and management issues most strongly predicted participants’ burnout [14]. Furthermore, many studies verified the key value of connections with colleagues to job burnout, such as those related to interpersonal justice [15], interpersonal conflicts [16], and group noncooperation [17]. Therefore, relationships with colleagues play a central role in helping individuals produce high performance [18] and in decreasing the level of job burnout in organizations [19]. However, prior investigations only focused on the qualities of relationships, such as closeness and intimacy [20] and did not examine the position (influence) of individuals in relational structures, that is, they overlooked the connectedness of workers involved in social networks.

Sociologists claimed that an individual’s interpersonal relationships can behave as if in a social network, and the extent to which an individual’s influence in the network is his “connectedness” as measured through the degree centrality index using social network analysis (SNA). Individuals with a high level of connectedness are in the central location in their social networks and are more influential in organizations [21]. According to different occupational relationships, two kinds of social networks exist in organizations—advisory and friendship networks [22]. Advisory networks are formed according to the need for job exchanges, whereas friendship networks arise from personal emotion. In terms of PHC workers, numerous studies proved that PHC workers’ behaviors were affected by the connectedness of their social networks [23]. Job burnout can also be seen as a behavior. Hence, we hypothesize that the connectedness of PHC workers involved in social networks can affect their job burnout. The present study attempts to identify the connectedness of PHC workers in rural China using SNA and explore the effects on their job burnout. Scientific evidence can help address job burnout effectively and improve the qualities of PHC in rural China.

## Methods

### Study design and sample

A cross-sectional survey study was conducted from December 2016 to March 2017. The convenience sampling method was used in this research, particularly the data requirements according to SNA. First, sample areas were selected on the basis of the cooperation foundation that was already established with the research group. Six counties were selected, namely, Zhijiang, Zaoyang, and Suizhou in Hubei Province, and Tanghe, Xixian, and Huaibin in Henan Province. Six counties were located in central China. Second, three to five township hospitals were selected from each of the sample areas according to their performance ranking in the previous year: “high,” “middle,” and “low” ranks. A total of 21 township hospitals were selected as sample hospitals. Third, sample populations were determined through the preliminary screening of the rosters of all personnel in each sample hospital and by considering all the people involved in the PHC services, including clinicians, nurses, public health workers, and village doctors. Each sample hospital had 30–35 respondents (average number: 33). A total of 663 PHC workers completed the survey. The response rate was 96.0% (663/690).

All participants were convened to complete the questionnaires. Questions, response choices, and academic investigations were explained to them before they filled in the questionnaires. The names of sample populations were replaced by numbers and were included in the lists distributed to each participant in order to investigate the social network of workers. Communicating with each other while filling out the questionnaires was forbidden to ensure the quality of the network data. The Cronbach’s alpha coefficient and average variance extracted (AVE) values were 0.849 and 0.552, respectively. The reliability and validity of the questionnaire were deemed acceptable because the Cronbach’s alpha coefficient and AVE values exceeded 0.7 and 0.5, respectively.

### Data measures

The dependent variable was the level of job burnout, which involved three dimensions: emotional exhaustion, depersonalization, and lack of personal accomplishment [24]. This was measured by the Maslach Burnout Inventory consisting of 22 items. Each item was scored from 0 to 6 on the self-reported frequency of the feeling addressed by each item. The emotional exhaustion domain consisted of nine items for a total score range of 0–54. Scores below 19, 19–26, and above 26 respectively indicated low, middle, and high levels of emotional exhaustion. The depersonalization domain consisted of five items for a total score range of 0–30. Scores below 6, 6–9, and above 9 indicated low, middle, and high levels of depersonalization, respectively. The personal accomplishment domain consisted of eight items for a total score range of 0–48. Score below 34, 34–39, and above 39 respectively indicated high, middle, and low levels of lack of personal accomplishment.

The independent variable was the connectedness of PHC workers in their advisory and friendship networks. The following questions measured the connectedness of advisory networks [25]: (1) Which colleagues will you consult when you encounter difficulties in your work? (2) Which colleagues will take the initiative to guide you when you encounter difficulties in your work? To measure the connectedness of friendship networks, the following queries were investigated: (1) Who are your close friends in private in addition to formal colleague relationships? (2) Which colleagues can help you keep secrets? Connectedness in networks was then measured using the degree centrality index of the SNA. The connectedness in the workers’ advisory and friendship networks was divided into high, middle, and low levels based on the average scores of degree centralities (advisory networks: 9.57 ± 11.40, friendship networks:7.95 ± 8.16). Individuals with a high level of connectedness are those who are in the central location in their social networks and are more influential in their organizations. Individuals with low connectedness levels include those who are at the edge of their social networks and are less influential in their organizations. Individuals with a middle level of connectedness are between the central and edge locations in their social networks and are commonly influential in their organizations. Degree centrality index was calculated using Formula (1). Standardizing the values for comparing degree centrality (Formula [2]) is necessary.

1 $$ {C}_D\left({n}_i\right)=d\left({n}_i\right)=\sum \limits_j{X}_{ij}, $$

2 $$ {C}_D^{\prime}\left({n}_i\right)=d\left({n}_i\right)/\left(g-1\right). $$

*X*_*ij*_ = relationship between *I* and *J*. If a relationship exists, then *X*_*ij*_ = 1, Otherwise, *X*_*ij*_ = 0.

*g*=the total number of people in the network.

The control variables comprised factors that might influence the results of connectedness acting on job burnout, including (1) types of work, (2) age, (3) gender, (4) educational background, (5) work experience (in years), (6) monthly income, and (7) daily work hours. The questionnaire used in this study is provided as Additional File 1.

### Statistical analysis

All data were independently double-entered and validated using EpiData. All statistical analyses were performed with SPSS. The degree centrality index was calculated with UCINET. All variables were presented as frequency distribution and percentage. The chi-square test was employed to investigate the corresponding associations of job burnout with the independent and control variables. Only variables with statistically significant differences were included in the cumulative logistic regression model. Values with *P* < 0.05 (two tailed) were considered statistically significant.

## Results

### Sample characteristics

Table 1 lists the characteristics of the sample population. Four types of PHC workers were included: doctors, nurses, public health workers, and village doctors. Nearly half of the respondents were village doctors (49.0%), followed by doctors (19.8%), nurses (15.8%), and public health personnel (15.4%). Females and males accounted for 44.5 and 55.5% of the respondents, respectively. Most participants were less than 46 years old (65.5%) and had a college degree and below (81.3%). Less than half of the rural PHC workers had fewer than 11 years of working experience (35.0%). More than half of the participants earned 2001–4000 RMB every month (53.8%) and worked 8–10 h every day (60.2%).

**Table 1:** Socio-demographic, connectedness in networks and job burnout level of PHC workers in rural China

Characteristics		Frequency	Percentage%
Types of work	Doctors	131	19.8
	Nurses	105	15.8
	Public health workers	102	15,4
	Village doctors	325	49.0
Gender	Male	368	55.5
	Female	295	44.5
Age	≤35	182	27.5
	36–45	252	38.0
	46–55	157	23.7
	≥56	72	10.9
Educational Background	Undergraduate Degree and Above	124	18.7
	College Degree	322	48.6
	High School and Below	217	32.7
Work Experiences (years)	≤10	232	35.0
	11–20	193	29.1
	≥21	238	35.9
Monthly Income	≤2000	219	33.0
	2001–4000	357	53.8
	≥4001	87	13.1
Daily Work Hours	≤7 h	55	8.3
	8-10 h	399	60.2
	≥11 h	209	31.5
Connectedness in Advisory Networks	Low	74	11.2
	Middle	467	70.4
	High	122	18.4
Connectedness in Friendship Networks	Low	109	16.4
	Middle	466	70.3
	High	88	13.3
Emotional Exhaustion	Low	406	61.2
	Middle	97	14.6
	High	160	24.1
Depersonalization	Low	510	76.9
	Middle	49	7.4
	High	104	15.7
Lack of Personal Accomplishment	Low	328	49.5
	Middle	105	15.8
	High	230	34.7

### Connectedness in social networks and the job burnout situation of PHC workers

Social networks included advisory and friendship networks. More than half of the participants were in the middle connectedness level in the advisory (70.4%) and friendship (70.3%) networks. In terms of job burnout, the emotional exhaustion (61.2%) and depersonalization (76.9%) of rural PHC workers were mainly in the low group. However, the low level of lack of personal accomplishment only accounted for 49.5% of respondents (Table 1).

### Predictors affecting the influence of connectedness in social networks on job burnout among PHC workers

A significant difference was found between the emotional exhaustion/depersonalization/lack of personal accomplishment levels on various control variables. The male respondents (*χ*^*2*^ = 12.037, *P* = 0.002) who had longer daily work hours (*χ*^*2*^ = 14.163, *P* = 0.007) seemed to have a high level of emotional exhaustion (Table 2). Rural PHC workers with a high level of educational background (*χ*^*2*^ = 14.193, *P* = 0.007) and had less work experiences (*χ*^*2*^ = 17.9743, *P* = 0.001) seemed to have a high level of depersonalization (Table 3). The lack of personal accomplishment of the different professional PHC workers varied (*χ*^*2*^ = 15.391, *P* = 0.017). Meanwhile, respondents with a high level of educational background (*χ*^*2*^ = 13.429, *P* = 0.009) and had less work experiences (*χ*^*2*^ = 15.614, *P* = 0.004) seemed to have a high level of lack of personal accomplishment (Table 4).

**Table 2:** Correlations between emotional exhaustion level, and demographic, connectedness in networks of PHC workers

Characteristics		Low	Middle	High	***χ***^***2***^	***P***
		N(%)				
Types of work	Doctors	83 (63.4)	19 (14.5)	29 (22.1)	4.005	0.676
	Nurses	60 (57.1)	19 (18.1)	26 (24.8)		
	Public health workers	69 (67.6)	13 (12.7)	20 (19.6)		
	Village doctors	194 (59.7)	46 (14.2)	85 (26.2)		
Gender	Male	230 (62.5)	39 (10.6)	99 (26.9)	12.037	0.002
	Female	176 (59.7)	58 (19.7)	61 (20.7)		
Age	≤35	99 (54.4)	30 (16.5)	53 (29.1)	7.516	0.276
	36–45	164 (65.1)	34 (13.5)	54 (21.4)		
	46–55	97 (61.8)	26 (16.6)	34 (21.7)		
	≥56	46 (63.9)	7 (9.7)	19 (26.4)		
Educational Background	Undergraduate Degree and Above	68 (54.8)	21 (16.9)	35 (28.2)	4.399	0.355
	College Degree	204 (63.4)	49 (15.2)	69 (21.4)		
	High School and Below	134 (61.8)	27 (12.4)	56 (25.8)		
Work Experiences (Years)	≤10	129 (55.6)	40 (17.2)	63 (27.2)	5.107	0.276
	11–20	124 (64.2)	24 (12.4)	45 (23.3)		
	≥21	153 (64.3)	33 (13.9)	52 (21.8)		
Monthly Income	≤2000	127 (58.0)	37 (16.9)	55 (25.1)	3.496	0.478
	2001–4000	229 (64.1)	45 (12.6)	83 (23.2)		
	≥4001	50 (57.5)	15 (17.2)	22 (25.3)		
Daily Work Hours	≤7 h	42 (76.4)	8 (14.5)	5 (9.1)	14.163	0.007
	8-10 h	252 (63.2)	57 (14.3)	90 (22.6)		
	≥11 h	112 (53.6)	32 (15.3)	65 (31.1)		
Connectedness in Advisory Networks	Low	45 (60.8)	4 (5.4)	25 (33.8)	9.469	0.050
	Middle	289 (61.9)	73 (15.6)	105 (22.5)		
	High	72 (59.0)	20 (16.4)	30 (24.6)		
Connectedness in Friendship Networks	Low	65 (59.6)	5 (4.6)	39 (35.8)	18.613	0.001
	Middle	281 (60.3)	81 (17.4)	104 (22.3)		
	High	60 (68.2)	11 (12.5)	17 (19.3)

**Table 3:** Correlations between depersonalization level, and demographic, connectedness in networks of PHC workers

Characteristics		Low	Middle	High	***χ***^***2***^	***P***
		N(%)				
Types of work	Doctors	94 (71.8)	11 (8.4)	26 (19.8)	5.505	0.481
	Nurses	81 (77.1)	7 (6.7)	17 (16.2)		
	Public health workers	86 (84.3)	6 (5.9)	10 (9.8)		
	Village doctors	249 (76.6)	25 (7.7)	51 (15.7)		
Gender	Male	285 (77.4)	22 (6.0)	61 (16.6)	2.679	0.262
	Female	225 (76.3)	27 (9.2)	43 (14.6)		
Age	≤35	128 (70.3)	16 (8.8)	38 (20.9)	6.769	0.343
	36–45	198 (78.6)	17 (6.7)	37 (14.7)		
	46–55	126 (80.3)	11 (7.0)	20 (12.7)		
	≥56	58 (80.6)	5 (6.9)	9 (12.5)		
Educational Background	Undergraduate Degree and Above	84 (67.7)	8 (6.5)	32 (25.8)	14.193	0.007
	College Degree	252 (78.3)	29 (9.0)	41 (12.7)		
	High School and Below	174 (80.2)	12 (5.5)	31 (14.3)		
Work Experiences (Years)	≤10	157 (67.7)	22 (9.5)	53 (22.8)	17.974	0.001
	11–20	158 (81.9)	11 (5.7)	24 (12.4)		
	≥21	195 (81.9)	16 (6.7)	27 (11.3)		
Monthly Income	≤2000	173 (79.0)	14 (6.4)	32 (14.6)	1.995	0.737
	2001–4000	274 (76.8)	26 (7.3)	57 (16.0)		
	≥4001	63 (72.4)	9 (10.3)	15 (17.2)		
Daily Work Hours	≤7 h	45 (81.8)	5 (9.1)	5 (9.1)	3.487	0.480
	8-10 h	311 (77.9)	27 (6.8)	61 (15.3)		
	≥11 h	154 (73.7)	17 (8.1)	38 (18.2)		
Connectedness in Advisory Networks	Low	53 (71.6)	1 (1.4)	20 (27.0)	18.020	0.001
	Middle	352 (75.4)	43(9.2)	72 (15.4)		
	High	105 (86.1)	5 (4.1)	12 (9.8)		
Connectedness in Friendship Networks	Low	77 (70.6)	5 (4.6)	27 (24.8)	9.955	0.041
	Middle	361 (77.5)	39 (8.4)	66 (14.2)		
	High	72 (81.8)	5 (5.7)	11 (12.5)

**Table 4:** Correlations between lack of personal accomplishment level, and demographic, connectedness in networks of PHC workers

Characteristics		Low	Middle	High	***χ***^***2***^	***P***
		N(%)				
Types of work	Doctors	54 (41.2)	17 (13.0)	60 (45.8)	15.391	0.017
	Nurses	50 (47.6)	13 (12.4)	42 (40.0)		
	Public health workers	48 (47.1)	22 (21.6)	32 (31.4)		
	Village doctors	176 (54.2)	53 (16.3)	96 (29.5)		
Gender	Male	183 (49.7)	58 (15.8)	127 (34.5)	0.022	0.989
	Female	145 (49.2)	47 (15.9)	103 (34.9)		
Age	≤35	76 (41.8)	33 (18.1)	73 (40.1)	7.635	0.266
	36–45	134 (53.2)	39 (15.5)	79 (31.3)		
	46–55	81 (51.6)	20 (12.7)	56 (35.7)		
	≥56	37 (51.4)	13 (18.1)	22 (30.6)		
Educational Background	Undergraduate Degree and Above	48 (38.7)	17 (13.7)	59 (47.6)	13.426	0.009
	College Degree	159 (49.4)	55 (17.1)	108 (33.5)		
	High School and Below	121 (55.8)	33 (15.2)	63 (29.0)		
Work Experiences (Years)	≤10	93 (40.1)	43 (18.5)	96 (41.4)	15.614	0.004
	11–20	97 (50.3)	28 (14.5)	68 (35.2)		
	≥21	138 (58.0)	34 (14.3)	66 (27.7)		
Monthly Income	≤2000	120 (54.8)	34 (15.5)	65 (29.7)	4.717	0.318
	2001–4000	169 (47.3)	58 (16.2)	130 (36.4)		
	≥4001	39 (44.8)	13 (14.9)	35 (40.2)		
Daily Work Hours	≤7 h	25 (45.5)	10 (18.2)	20 (36.4)	0.866	0.929
	8-10 h	202 (50.6)	60 (15.0)	137 (34.3)		
	≥11 h	101 (48.3)	35 (16.7)	73 (34.9)		
Connectedness in Advisory Networks	Low	26 (35.1)	9 (12.2)	39 (52.7)	22.744	0.000
	Middle	232 (49.7)	69 (14.8)	166 (35.5)		
	High	70 (57.4)	27 (22.1)	25 (20.5)		
Connectedness in Friendship Networks	Low	43 (39.4)	16 (14.7)	50 (45.9)	12.030	0.017
	Middle	232 (49.8)	74 (15.9)	160 (34.3)		
	High	53 (60.2)	15 (17.0)	20 (22.7)		

After discovering that gender and daily work hours were significantly associated with emotional exhaustion, educational background and work experiences were revealed to be significantly associated with depersonalization and lack of personal accomplishment. Moreover, the type of work was significantly associated with the lack of personal accomplishment. These factors were controlled using cumulative logistic regression analysis to examine the impact of connectedness in social networks on the workers’ job burnout.

As shown in Table 5, connectedness level in friendship networks had a significant impact on emotional exhaustion. Compared with PHC workers who had a high level of connectedness in friendship networks, participants with a low level of connectedness in friendship networks seemed to have an increased degree of emotional exhaustion (*β* = 0.769, 95% *CI* = 0.080–1.458, *P* = 0.029). Table 6 indicates that the connectedness degree in advisory networks had a significant impact on depersonalization. The respondents in the middle level of connectedness in advisory networks had a higher level of depersonalization than a high level of connectedness (*β* = 0.739, 95% *CI* = 0.130–1.348, *P* = 0.017). Table 7 reveals that the connectedness degree in social networks had a significant impact on the workers’ lack of personal accomplishment. Respondents in the low (*β* = 1.042, 95% *CI* = 0.309–1.775, *P* = 0.005) and middle (*β* = 0.583, 95% *CI* = 0.111–1.055, *P* = 0.015) levels of connectedness in their advisory networks had a higher level of lack of accomplishment than a high level of connectedness. Otherwise, the results showed that doctors (*β* = 0.748, 95% *CI* = 0.304–1.192, *P* = 0.001) and public health workers (*β* = 0.523, 95% *CI* = 0.017–1.029, *P* = 0.043) in township hospitals had a higher level of lack of personal accomplishment than village doctors.

**Table 5:** Outcome of a cumulative logistic regression model examining control variables, connectedness in networks with emotional exhaustion

Characteristics		Reference	***B***	***P***	***OR***	95%***CI***	
						Lower	Upper
**Control Variables**	**Gender**						
	Male	Female	−0.053	0.742	0.948	−0.369	0.263
	**Daily Work Hours**						
	≤7 h	≥11 h	−1.165	0.001	0.312	−1.856	−0.475
	8-10 h		−0.484	0.005	0.616	−0.822	−0.145
**Connectedness in Advisory Networks**	Low	High	−0.382	0.283	0.682	−1.081	0.316
	Middle		−0.401	0.081	0.669	−0.851	0.050
**Connectedness in Friendship Networks**	Low	High	0.769	0.029	2.157	0.080	1.458
	Middle		0.399	0.137	1.490	− 0.127	0.925

**Table 6:** Outcome of a cumulative logistic regression model examining control variables, connectedness in networks with depersonalization

Characteristics		Reference	***B***	***P***	***OR***	95%***CI***	
						Lower	Upper
**Control Variables**	**Educational Background**						
	Undergraduate Degree and Above	High School and Below	0.439	0.107	1.551	−0.094	0.072
	College Degree		−0.088	0.699	0.915	−0.536	0.359
	**Work Experiences (Years)**						
	≤10	≥21	0.794	0.001	2.212	0.337	1.251
	11–20		0.021	0.934	1.021	−0.484	0.527
**Connectedness in Advisory Networks**	Low	High	0.579	0.194	1.784	−0.294	1.453
	Middle		0.739	0.017	2.093	0.130	1.348
**Connectedness in Friendship Networks**	Low	High	0.206	0.619	1.228	−0.606	1.018
	Middle		−0.090	0.784	0.914	−0.733	0.552

**Table 7:** Outcome of a cumulative logistic regression model examining control variables, connectedness in networks with lack of personal accomplishment

Characteristics		Reference	***B***	***P***	***OR***	95%***CI***	
						Lower	Upper
**Control Variables**	**Types of work**						
	Doctors	Village Doctors	0.748	0.001	2.112	0.304	1.192
	Nurses		0.365	0.107	1.440	−0.079	0.809
	Public Health Workers		0.523	0.043	1.687	0.017	1.029
	**Educational Background**						
	Undergraduate Degree and Above	High School and Below	0.214	0.376	1.238	−0.260	0.688
	College Degree		−0.036	0.845	0.964	−0.395	0.323
	**Work Experiences (Years)**						
	≤10	≥21	0.446	0.022	1.562	0.063	0.829
	11–20		0.217	0.268	1.242	−0.167	0.600
**Connectedness in Advisory Networks**	Low	High	1.042	0.005	2.834	0.309	1.775
	Middle		0.583	0.015	1.791	0.111	1.055
**Connectedness in Friendship Networks**	Low	High	0.607	0.074	1.834	−0.060	1.273
	Middle		0.375	0.145	1.454	−0.129	0.879

## Discussion

This study demonstrated that 24.1, 15.7, and 34.7% of PHC workers in rural China had high levels of emotional exhaustion, depersonalization, and lack of personal accomplishment, respectively. Overall, serious job burnout of rural PHC workers in China still exists. These findings are similar with the conclusions reported in previous studies about the burnout symptom of PHC workers [8]. With the implementation of the national health coverage strategy, the workload of PHC workers in China is gradually increasing. Specifically, only 8.3% PHC workers had reasonable working times of less than 8 h a day. Respondents who had longer daily work hours reported high levels of emotional exhaustion. Meanwhile, rural PHC workers who had less work experiences experienced high levels of depersonalization and lack of personal accomplishment. On the one hand, physicians with few work years had a high level of lack of personal accomplishment due to the lack of respect from patients [26]. On the other hand, PHC workers with less work experiences had more single content health services compared with workers who had longer work years, so they had a high level of depersonalization. In addition, compared with village doctors, physicians and public health workers in township hospitals had higher levels of lack of personal accomplishment. These results can be related to the relationship between the different types of PHC workers and service objects. The relationship between village doctors and residents was closer and the influence among residents was stronger compared with the workers in township hospitals. Hence, village doctors seemed to have a high level of personal accomplishment. At the same time, this outcome also showed the important influence of interpersonal relationship on job burnout.

The majority of PHC workers involved in the social networks in rural China were in the high and middle levels of connectedness. Thus, the connection and cooperation among different types of PHC professionals was gradually strengthening through the implementation of a team model to provide health services. Compared with the previous independent health service delivery model, the team model promotes the integration of medical intervention and prevention and strengthens the establishment of the three-tiered rural health network in China [27]. However, 11.2 and 16.4% of the PHC workers were still in the low level of connectedness in advisory networks and friendship networks, respectively. The environment of inadequate PHC workers required us to pay attention to each PHC worker. Meanwhile, this research showed that the connectedness of PHC workers involved in social networks could affect their job burnout. Therefore, through the analysis of the relationship between the two types of connectedness in social network and the three dimensions of job burnout, this work could provide some suggestions to alleviate the problem of job burnout of PHC workers.

Results of the cumulative regression model revealed that an individual with a low level of connectedness in friendship networks had serious emotional exhaustion. Two reasons were found for such an outcome. First, personal friendship can help complete the job and reduce the pressure on PHC workers, especially in rural China. In terms of physicians, one chief of public health said: “*Some doctors were reluctant to do public health works because of their heavy workload. So I could only convince him through the identity of a friend, not force him to do it.*” As for public health workers, some public health tasks (such as conducting health follow-up) are completed because of the friendship with village doctors resulting from the close connections between such doctors and residents [28]. Therefore, the pressure of completing work and the emotional exhaustion of PHC workers can be relieved through the establishment of friendships with other employees. Second, the establishment of friendship networks can help PHC workers have a homogeneous working atmosphere and environment so as to reduce emotional exhaustion. The previous study showed that a high degree of connectedness involving friendship networks enhances the dissemination of knowledge, imitation of behaviors, and related social processes, thereby resulting in the homogeneity of the cultural atmosphere and practice patterns [18]. Moreover, in a relatively relaxed working environment, PHC workers can talk about their work problems and improve their abilities with help from colleagues, hence they have low level of emotional exhaustion. Therefore, a high connectedness level of rural PHC workers in friendship networks could mitigate the emotional exhaustion of working, thus supporting the results of this study. However, informal communication paths (friendship) can be difficult to supervise and manage within organizations. Hence, leaders should pay attention to the correct guidance of communication behavior.

In addition, the research findings indicated that PHC workers with a low level of connectedness in advisory networks seemed to have a high level of depersonalization and lack of personal accomplishment. Depersonalization means feelings of callousness in treating colleagues or service objects [29]. Hence, such individuals do not exhibit enthusiasm about anything. From the perspective of social capital, a person with social relationships can acquire knowledge and information, and such capability can be translated into the individual’s abilities [30]. Therefore, a high connectedness level in advisory networks means that individuals have strong working abilities. People with competence usually have a positive attitude toward the job according to the “knowledge–attitude–practice” model [31]. It was logical that PHC workers who had a high level of connectedness involved in advisory networks also had a high degree of working abilities, a positive attitude, and a low level of depersonalization. Meanwhile, PHC workers with a high level of connectedness involved in advisory networks were prone to have personal accomplishments. A previous research posited that individuals with high connectedness reflected a high degree of individual prestige and influence ability among colleagues [32]. PHC workers have psychological needs for social respect. This can explain the previous conclusion that the level of lack of personal accomplishment of village doctors was lower than that of doctors and public health workers in township hospitals because the former had more individual prestige and influence ability among residents compared with their counterparts in townships. Furthermore, PHC workers with a high level of connectedness are located at the center of advisory networks; hence, their behaviors and ideas could have a strong influence on others, even in organizations. The level of burnout is no exception. Managers of rural health institutions should consider individuals with a high level of connectedness in advisory networks as core staff of the team [33] and establish an incentive mechanism for these core members. Core workers with lower burnout level in the organizations can influence and even reduce the overall burnout level of PHC workers.

This study had several limitations. First, these samples might not represent the conditions of all rural PHC workers because the convenience sampling method was used. Second, this research analyzed that social network affected the job burnout of PHC workers in rural China but only through using the degree centrality index. Indexes could reflect the structural characteristics of social networks, which may also influence the job burnout level of rural PHC workers. Third, this study took the connectedness of PHC workers in their advisory and friendship networks into consideration, while it ignored family network. Thus, further works must be conducted to expand the knowledge in this area.

## Conclusions

PHC workers in rural China face a severe problem of job burnout. The workers’ level of emotional exhaustion was affected by the daily working hours beyond the reasonable range. The problem of depersonalization and lack of personal accomplishment of less experienced personnel, doctors, and public health workers in township hospitals should be addressed further in the future. Meanwhile, individuals with a low level of connectedness in friendship networks had serious emotional exhaustion, thereby indicating the importance of establishing and strengthening organizational culture. Organizations should pay more attention to informal communication networks among staff, establish an informal communication platform and information feedback mechanism, promote and manage friendship networks, and help PHC workers relieve the problem of emotional exhaustion. Meanwhile, PHC workers with low connectedness level of advisory networks had a high level of depersonalization and lack of personal accomplishment. Thus, managers should advance the core position of individuals with a high level of connectedness in advisory networks and have them play the role of “opinion leader” so that they can help others overcome burnout symptoms.

## Supplementary information

**Additional file 1.** Questionnaire on Social Network and Job Burnout of Primary Health Care Workers in Rural

## Data Availability

The datasets used and/or analyzed during the current study are available from the corresponding author on reasonable request. Contact information: zcfeng@hust.edu.cn.

## Abbreviations

PHC: Primary Health Care
N: Number
P: P-Value
B: Beta
OR: Odds Ratio
CI: Confidence Interval
