# Supplementary material for: Could the connectedness of primary health care workers involved in social networks affect their job burnout? A cross-sectional study in six counties, Central China
Source: BMC Health Serv Res. 2020 Jun 18;20:557. doi: 10.1186/s12913-020-05426-9 (PMC7302340; doi:10.1186/s12913-020-05426-9)
Supplement: Supplementary file 1 — Additional file 1. Questionnaire on Social Network and Job Burnout of Primary Health Care Workers in Rural [file 12913_2020_5426_MOESM1_ESM.docx]

**Questionnaire on Social Network and Job Burnout of Primary Health Care Workers in Rural**

Dear PHC workers:

we are the research group of School of medicine and health management, Tongji Medical College, Huazhong University of science and technology. The purpose of this survey is to understand the social network and job burnout of PHC workers in rural areas of China. We guarantee that the survey data is only used for academic research and will not disclose your relevant information at any time.

Thank you for your support and cooperation.

**Part one**

Please refer to the personnel list on the last page and list the personnel number according to the following questions.

(1) Which colleagues will you consult when you encounter difficulties in your work?

(2) Which colleagues will take the initiative to guide you when you encounter difficulties in your work?

(3) Who are your close friends in private in addition to formal colleague relationships?

(4) Which colleagues can help you keep secrets?

**Part two**

Please read the following items carefully, and mark "√" on the corresponding number according to the conformity degree between the description and your actual situation.

0 = completely inconsistent, 1 = not consistent, 2 = relatively inconsistent, 3 = general, 4 = relatively consistent, 5 = consistent, 6 = fully compliant.

| Items | 0 | 1 | 2 | 3 | 4 | 5 | 6 |
| --- | --- | --- | --- | --- | --- | --- | --- |
| A1 I feel that work has a great influence on my mood |  |  |  |  |  |  |  |
| A2 After work, I feel exhausted. |  |  |  |  |  |  |  |
| A3After getting up in the morning, I feel very tired, but I have to face a new day. |  |  |  |  |  |  |  |
| A4 Work makes me tired. |  |  |  |  |  |  |  |
| A5 The work depressed me. |  |  |  |  |  |  |  |
| A6 I feel like I'm paying too much at work. |  |  |  |  |  |  |  |
| A7 Dealing with people all day makes me feel nervous. |  |  |  |  |  |  |  |
| A8 It's easy to create pressure when dealing directly with people. |  |  |  |  |  |  |  |
| A9 I feel like I'm exhausted. |  |  |  |  |  |  |  |
| B1 Sometimes I feel like I treat my patients like inanimate objects. |  |  |  |  |  |  |  |
| B2 After being a doctor, I felt numb. |  |  |  |  |  |  |  |
| B3 I'm afraid that work will make me cold. |  |  |  |  |  |  |  |
| B4 I will be indifferent to some patients. |  |  |  |  |  |  |  |
| B5 Sometimes I think it's the patient's own problem, and he blames me instead. |  |  |  |  |  |  |  |
| C1 It's easy for me to understand the patient's feelings. |  |  |  |  |  |  |  |
| C2 I can deal with the patient's problems very well. |  |  |  |  |  |  |  |
| C3 I feel that work enables me to positively influence or improve the lives of others. |  |  |  |  |  |  |  |
| C4 I feel energetic. |  |  |  |  |  |  |  |
| C5 It's easy for me to build rapport with patients. |  |  |  |  |  |  |  |
| C6 I feel very happy to be in close contact with the patient. |  |  |  |  |  |  |  |
| C7 I feel that I have made a lot of achievements in my work. |  |  |  |  |  |  |  |
| C8 At work, I can deal with emotional problems calmly. |  |  |  |  |  |  |  |

**Part three**

(1) Number:

(2) Gender: male / female

(3) Age:

(4) Educational background: undergraduate degree and above / college Degree /

high school and below

(5) Work experience (in years):

(6) Monthly income: RMB

(7) Daily work hours:

**Thank you again for your cooperation and best wishes for you.**
